# Long-term Follow-up 15 Years After Duodenal Switch or Gastric Bypass for Super Obesity: a Randomized Controlled Trial

**DOI:** 10.1007/s11695-023-06767-0

**Published:** 2023-08-16

**Authors:** Filip Möller, Jakob Hedberg, Martin Skogar, Magnus Sundbom

**Affiliations:** Department of Surgical Sciences, Uppsala University, Entrance 70, 751 85 Uppsala, Sweden

**Keywords:** Roux-en-Y gastric bypass, Biliopancreatic diversion with duodenal switch, Long-term results, Obesity, Weight loss, Diabetes mellitus, BAROS, Bariatric surgery

## Abstract

**Background:**

In super obesity, Roux-en-Y gastric bypass (RYGB) may be insufficient why some surgeons advocate biliopancreatic diversion with duodenal switch (BPD/DS), a more malabsorptive procedure. There is a paucity of evidence regarding results beyond 10 years, especially after BPD/DS. The aim of this randomized controlled trial was to compare the long-term outcome of BPD/DS, and RYGB in patients with super obesity, i.e., body mass index (BMI) > 50 kg/m^2^.

**Methods:**

This is a 13- to 17-year follow-up study of a single-center, single-blinded randomized trial in which 47 patients (BMI > 48 and eligible for bariatric surgery) were randomized 1:1 to BPD/DS and RYGB (25 men, 24 BPD/DS, 39.1 ± 9.9 years, BMI 54.5 ± 6.1 kg/m^2^). The primary outcome was weight loss. The study was financed by Swedish governmental funding of clinical research (ALF). Trial registration number: ISRCTN10940791.

**Results:**

Thirty-four (18 BPD/DS) of the living 42 patients (81.0%) participated. BPD/DS resulted in higher BMI loss (20.4 ± 7.9 vs. 12.4 ± 8.6, *p* = .008) and higher percent of total body weight loss (37.5% ± 12.2 vs. 22.8% ± 14.8, *p* = .004). BPD/DS was associated with lower fasting glucose, glycated hemoglobin (HbA1c), and low-density lipoprotein (LDL) as well as lower hemoglobin. Adverse events were more common after BPD/DS (2.7 vs. 0.9 per patient, *p* = .004). The global assessment tool BAROS (Bariatric Analysis and Reporting Outcome System) demonstrated superior scores for BPD/DS (*p* = .047).

**Conclusion:**

When compared to RYGB, BPD/DS results in superior weight loss and metabolic control as well as superior BAROS score, however, at the cost of more adverse events.

**Graphical Abstract:**

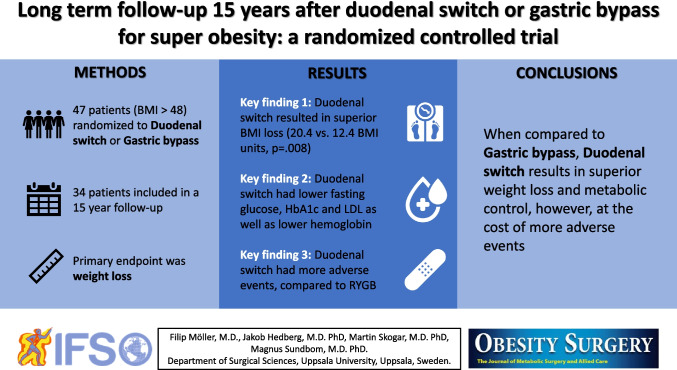

## Introduction

Obesity and obesity-related diseases are rapidly increasing worldwide [1]. In addition to big health risks and negative impact on quality of life [2], obesity is a major economic burden with 69 billion dollars spent on health care in the USA alone in 2013 [3]. In 2017, 42.4% of US adults had obesity (Body Mass Index, BMI > 30) and 9.2% were having severely obesity (BMI > 40) [4, 5]. Patients with super obesity (BMI > 50) have been increasing disproportionally and pose an added challenge due to more serious comorbidities and technical difficulties during surgery [6, 7].

There is currently no consensus on the preferred surgical procedure in patients with super obesity. Roux-en-Y gastric bypass (RYGB), often referred to as golden standard among bariatric procedures, has excellent results on weight-loss and comorbidities. RYGB can lead to insufficient weight loss in patients with super obesity [8, 9], and therefore, some surgeons advocate a more malabsorptive procedure, known to result in persistent weight loss and superior metabolic control [10–14]. These procedures include the biliopancreatic diversion, introduced by Scopinaro [15], and the biliopancreatic diversion with duodenal switch (BPD/DS), a modification by Hess [16] based on a bile-reflux reducing procedure by DeMeester [17]. However, BPD/DS is a technically more complex operation than RYGB and requires a more rigid follow-up program due to the risk of malnutritional deficiencies and various gastrointestinal adverse effects such as diarrhea, acid reflux, and foul-smelling flatus [12, 18]. In 2004–2007, we randomized 47 patients with super obesity to BPD/DS or RYGB. At three years, the expected results were found, i.e., greater weight loss and superior glucose homeostasis in BPD/DS, at the cost of diarrhea [19]. In a previous study including all our patients with super obesity (*n* = 211), BPD/DS was also found to have a superior score (4.7 vs. 4.0, *p* < 0.05) [11], when analyzed with the validated and well-established Bariatric Analysis and Reporting Outcome System (BAROS) [20, 21]. The global assessment tool BAROS judges weight loss, changes in comorbidities and quality of life, while points are deducted for complications and reoperations [22].

The aim of this study was to compare the long-term outcome of BPD/DS and RYGB beyond 10 years in a randomized controlled trial on super obesity by assessing weight loss, comorbidities, adverse events, quality of life, and biochemical profiles as well as patient-rated gastrointestinal symptoms and overall satisfaction.

## Methods

### Study Design and Participants

All 47 patients from our original single-center, single-blinded, randomized trial [19] (25 men, age 39.1 ± 9.9 years, BMI 54.5 ± 6.1 kg/m^2^) were eligible to join this long-term follow-up. Details of the study design have been described earlier [19], but in short, from 2004 to 2007, 99 patients referred to us for bariatric surgery with BMI > 48 were assessed. Inclusion criteria were BMI > 48, above 18 years of age and being referred to us for bariatric surgery, exclusion criteria were language difficulties, previous problems with diarrhea and suspected inflammatory bowel disease. 90 patients deemed eligible for randomization, nine of which were excluded on medical grounds or language difficulties. The 47 patients who accepted participation were stratified by gender and BMI (> 53 or < 53 kg/m^2^) and were randomly assigned 1:1 between BPD/DS and RYGB. A sample size of 80 patients was calculated necessary (30% improved weight loss, 5% significance, and 80% power) but increasing numbers of patients declining inclusion led to premature closure of inclusion. The type of procedure was unknown to the participants and ward staff until 2 days after the surgery. All patients provided informed written consent.

### Surgery and Postoperative Follow-up

Our operative technique has been described previously [19]. In short, both procedures were performed through an upper midline incision. BPD/DS constituted of a 36-Fr sleeve gastrectomy and a 150-cm alimentary limb, emptying into a 100-cm common limb, while the remaining small bowel formed the biliary limb. In RYGB, a small gastric pouch was anastomosed to a 120-cm Roux limb, using a 50-cm biliary limb. Postoperatively, patients were started on multivitamin supplementation (iron 15 mg, calcium 240 mg, vitamin A 600 µg, vitamin D3 750 µg, vitamin E 60 mg) and vitamin B_12_ injections. Following the second year, RYGB patients had annual checks-ups at their primary care physician, while BPD/DS patients continued their follow-up at the Department of Metabolic Medicine at our hospital.

### Outcomes

The primary outcomes were weight loss, and the secondary outcomes were change in comorbidities, adverse effects, biochemical profiles, patient-rated quality of life and gastrointestinal symptoms, overall satisfaction and the calculated BAROS score.

### Long-term Follow-up

Thirteen to seventeen (mean 15.4) years after the initial surgery, the included patients were asked to complete a questionnaire regarding current weight and comorbidities, present medication, subsequent surgeries, and gastrointestinal symptoms. In addition, they rated their overall satisfaction with the procedure (very satisfied, satisfied, dissatisfied, and very dissatisfied), and if they would recommend their procedure to a friend seeking bariatric surgery. For BAROS scoring, we included a translated version of the Moorhead-Ardelt Quality of life Questionnaire (MAQ), grading six aspects of quality of life on a 10-point Likert scale [21]. Weight was self-reported and is presented as change in body mass index (BMI) as well as percent total body weight loss (%TBWL). The following comorbidities were analyzed; diabetes; hypertension/cardiovascular disease, sleep apnea, and dyslipidemia. Presence of a comorbidity was defined as use of medication for the specific condition, or Continuous Positive Airway Pressure (CPAP) for sleep apnea, and remission was defined as complete cessation of medication or CPAP. Information regarding adverse effects and subsequent surgeries were collected from the questionnaires as well as from medical records. International Classification of Diseases 10th Revision (ICD-10) was used to aid identifying complications from medical records. The gastrointestinal symptoms (vomiting, reflux, dumping, abdominal pain, diarrhea, soling and foul-smelling gases) were rated by the patient as occurring daily, weekly, monthly, or yearly. According to the BAROS scoring tool; weight loss, improvement in medical conditions and quality of life were given up to 3 points each, while 1 and 0.2 points were deducted for major or minor complications and adverse events, respectively [22, 23]. The outcome of the procedure was classified as failure (≤ 1 point), fair (> 1 to 3 points), good (> 3 to 5 points), very good (> 5 to 7 points), or excellent (> 7 to 9 points) [22, 23]. Because of the long-term focus, we also calculated a BAROS score without including complications, as BAROS is mainly designed for perioperative complications, not for assessment of additional surgeries nor adverse effects several years after the bariatric procedure. Finally, biochemical profiles concerning hemoglobin, vitamin B12, folate, albumin, fasting glucose, hemoglobin A1c (HbA1c), low-density lipoprotein (LDL), high-density lipoprotein (HDL), and triglycerides were analyzed.

### Statistics

Regarding the frequency of gastrointestinal symptoms, the result was dichotomized into often (once per week or more) or seldom (less than once a week). For between-group comparisons of continuous variables, independent samples *t*-test was used for normally distributed data and the Mann–Whitney U test for non-normally distributed data. Chi^2^ test or Fischer’s exact test was used for categorical variables when comparing between groups, while McNemar test was used for comparisons within groups. A *p* value of < 0.05 was considered statistically significant. IBM® SPSS® 28 was used for the statistical analysis.

### Ethics

This study has been approved by the regional ethical review board (Dnr: 2014/318).

### Trial Registration

The trail has been registered at ISRCTN.com (Registration number: ISRCTN10940791).

### Funding

The study was financed by Swedish governmental funding of clinical research (ALF).

## Results

**Fig. 1:**
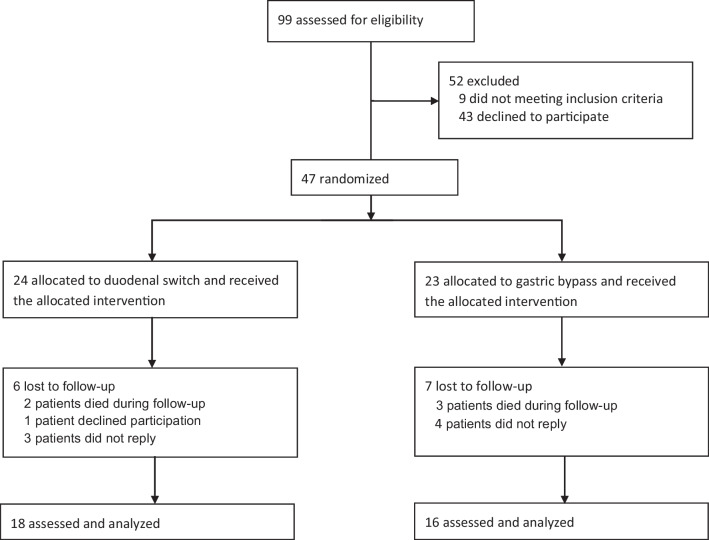
Participant flow through the study

Of the initial 47 patients (24 BPD/DS, 23 RYGB), 42 patients were still alive and 34 (81%) (18 BPD/DS, 16 RYGB) accepted participation and returned our questionnaires (Fig. 1). The median follow-up time from surgery was 15 years (range 13 to 17 years). At follow-up, mean age (38.3 ± 8.6 vs. 38.5 ± 9.3) and proportion of men (61.1%, *n* = 11 vs. 56.3%, *n* = 9) did not differ between the two groups (Table 1). In a sensitivity analysis, baseline characteristics (gender, type of surgery, and preoperative BMI) did not differ between the responding patients (*n* = 34) and those lost to follow-up (*n* = 13).

**Table 1:** Anthropometric data, comorbidities, and biochemical profile at baseline and long-term follow-up

	BPD/DS (*n* = 18)			RYGB (*n* = 16)			
	Preop	Long-term	*p* value^1^	Preop	Long-term	*p* value^1^	*p* value^2^
BMI kg/m^2^, mean ± SD	54.2 ± 7.1	33.8 ± 7.3	** < .001**^b^	53.8 ± 5.7	41.4 ± 8.6	** < .001**^b^	**.008**^a^
Age, mean ± SD	38.3 ± 8.6			38.5 ± 9.3			.945^a^
Men, % (n)		61.1 (11)			56.3 (9)		1.000^c^
Hypertension, % (n)	44.4 (8)	22.2 (4)	.219^d^	31.3 (5)	37.5 (6)	1.00^d^	.457^c^
Hyperlipidemia, % (n)	22.2 (4)	11.1 (2)	.625^d^	6.3 (1)	6.3 (1)	1.00^d^	1.00^c^
Diabetes, % (n)	22.2 (4)	0 (0)	.125^d^	18.8 (3)	12.5 (2)	1.00^d^	.214^c^
Sleep apnea, % (n)	22.2 (4)	5.6 (1)	.250^d^	12.5 (2)	0 (0)	.500^d^	1.000^c^
Hemoglobin^3^, mean ± SD	145.3 ± 10.1	122.4 ± 18.8	** < .001**^b^	140.6 ± 8.0	140.1 ± 6.2	.237^b^	**.002**^a^
Albumin^3^, mean ± SD	39.7 ± 2.2	36.3 ± 5.2	**.013**^b^	38.7 ± 2.4	38.2 ± 3.1	.656^b^	.274^a^
HbA1c^4^, mean ± SD	31.4 ± 10.4	30.8 ± 7.1	.839^b^	31.7 ± 12.5	41.8 ± 4.9	**.031**^b^	** < .001**^a^
Glucose^4^, mean ± SD	6.2 ± 1.4	5.1 ± 0.7	**.001**^b^	5.4 ± 0.8	6.2 ± 0.9	**.003**^b^	** < .001**^a^
LDL^4^, mean ± SD	3.0 ± 0.8	1.7 ± 0.7	** < .001**^b^	3.2 ± 0.8	2.8 ± 0.7	**.032**^b^	** < .001**^a^
HDL^4^, mean ± SD	1.1 ± 0.2	1.4 ± 0.7	.108^b^	1.1 ± 0.2	1.3 ± 0.4	.087^b^	.541^a^
Triglycerides^4^, mean ± SD	2.0 ± 1.0	0.9 ± 0.2	** < .001**^b^	2.0 ± 1.0	1.4 ± 0.8	.148^b^	.063^a^

^1^Within groups, i.e., before versus after surgery. ^2^Between groups in the long-term follow-up. ^3^g/l. ^4^mmol/l. ^a^Independent samples *t*-test. ^b^Paired samples *t*-test. ^c^Fischer’s exact test. ^d^McNemar’s test. *Preop*, preoperative; Long-term, after 13–17 years; *BMI*, body mass index; *BPD*/*DS*, biliopancreatic diversion with duodenal switch; *RYGB*, Roux-en-Y gastric bypass; *HbA1c*, glycated hemoglobin; *LDL*, low density lipoprotein; *HDL*, high density lipoprotein; *SD*, standard deviation. Bold indicates statistical significance

### Weight Loss

**Fig. 2:**
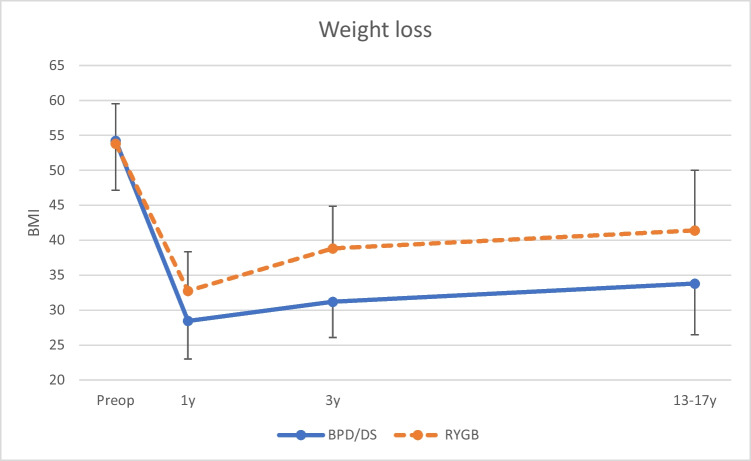
Weight loss, demonstrated as BMI at the actual time points. *BMI*, body mass index; *BPD/DS*, biliopancreatic diversion with duodenal switch; *RYGB*, Roux-en-Y gastric bypass; *Preop*, preoperative; *1y*,  1 year postoperative; *3y*, 3 years postoperative; *13–17y*, 13–17 years postoperative

The BPD/DS group had a significantly higher weight loss. The BMI at follow-up was 33.8 ± 7.3 vs. 41.4 ± 8.6, which corresponded to a total BMI loss of 20.4 ± 7.9 vs. 12.4 ± 8.6 and %TBWL of 37.5% ± 12.2 vs. 22.8% ± 14.8, for BPD/DS and RYGB, respectively (all *p* < 0.01) (Table 1 and Fig. 2). Notably, 53.3% (*n* = 8) of the patients in the RYGB group had a BMI > 40 and were thus still classified as having severe obesity.

### Comorbidities

There was no difference in comorbidities between the two groups at follow up. However, no BPD/DS-operated patient had diabetes mellitus at long-term follow-up (Table 1).

### Adverse Events and Mortality

Adverse events were more common in the BPD/DS group (2.7 vs. 0.9 per patient, *p* < 0.004) with anemia, vitamin/mineral deficiency, and symptomatic cholelithiasis, with or without biliary events, being the most common. Subsequent surgeries were numerically more common after BPD/DS (0.9 vs. 0.3 surgeries per patient, *p* = 0.070) but showed no statistical significance (Table 2). Three BPD/DS patients had significant hypoalbuminemia, leading to reversal surgery in one patient. Furthermore, two BPD/DS patients underwent emergency surgery due to small bowel perforation and one due to large bowel perforation (perforated diverticulitis). Despite this, the long-term mortality did not differ (2 BPD/DS vs. 3 RYGB). One patient in the BPD/DS group died of postoperative pulmonary embolism and one patient in the RYGB group died of severe swine flu; no data was available for the other mortalities.

**Table 2:** Registered number of adverse events, additional surgeries, and mortality during follow-up

	BPD/DS (*n* = 18)	RYGB (*n* = 16)	*p* value	Total (*n* = 34)
Early postoperative complications				
Postoperative bleeding	1	0	1.000^a^	1
Postoperative biliary leak	1	0	1.000^a^	1
Postoperative abscess	1	0	1.000^a^	1
Reoperation without pathology	0	1	.471^a^	1
Postoperative MI	1	0	1.000^a^	1
Additional surgery				
Cholecystectomy	6	1	.090^a^	7
Bowel perforation	3	0	.230^a^	3
Bowel obstruction	0	1	.471^a^	1
Incisional hernia repair	4	1	.340^a^	5
Reversal due to resistant hypoalbuminemia	1	0	1.000^a^	1
Deficiencies				
Anemia	12	4	**.020**^a^	16
Vitamin/mineral deficiency	8	4	.297^a^	12
Hypoalbuminemia	3	1	.604^a^	4
Non-surgical conditions				
Peptic ulcer disease/esophagitis	1	0	1.000^a^	1
Alcohol abuse	3	2	1.000^a^	5
Severe depression	1	2	.591^a^	3
Total number (per patient)	48 (2.7)	15 (0.9)	**.004**^b^	61 (1.9)
Total additional surgeries (per patient)	16 (0.9)	4 (0.3)	.070^b^	20 (0.6)
Patients with any adverse event (%)	17 (94)	9 (56)	.**014**^**a**^	26 (76)
30-day mortality (*n* = 47)	0	1		1
Long-term mortality (*n* = 47)	2	3		5

^a^Fischer’s exact test. ^b^Mann-Whitney *U* test. *BPD/DS*, biliopancreatic diversion with duodenal switch; *RYGB*, Roux-en-Y gastric bypass; *MI*, myocardial infarction. Bold indicates statistical significance

### Gastrointestinal Symptoms

Reflux was more common in the BPD/DS group (22.2% vs. 0%, *p* = 0.043) whereas no differences were seen among the remaining symptoms: vomiting, dumping, abdominal pain, diarrhea, soiling, and foul smelling flatus.

### Overall Satisfaction and Quality of Life

A high proportion of patients were satisfied or very satisfied with their procedure (BPD/DS: 72% and RYGB: 56%) and would recommend it to a friend seeking bariatric surgery (BPD/DS: 77.7% and RYGB: 66.6%), however without statistical significance between the two groups (*p* > 0.999 and *p* = 0.712, respectively). Quality of life (MAQ) was rated 0.50 ± 1.11 for BPD/DS and 0.24 ± 1.55 for RYGB (Table 3).

**Table 3:** BAROS scores

	BPD/DS (*n* = 18)	RYGB (*n* = 16)	*p* value
Weight loss sub-score	2.22 ± 0.81	1.31 ± 1.14	**.011**^a^
Comorbidity sub-score	0.83 ± 1.29	0.25 ± 1.18	.182^a^
Quality of life (MAQ)	0.50 ± 1.11	0.24 ± 1.55	.575^a^
Complication sub-score	− 0.58 ± 0.42	− 0.46 ± 0.45	.446^a^
BAROS score	2.98 ± 2.32	1.34 ± 2.30	**.047**^a^
BAROS w/o complication sub-score	3.55 ± 2.43	1.80 ± 2.20	**.035**^a^

^a^Independent samples *t*-test. *BAROS*, Bariatric Analysis and Reporting Outcome System; *BPD*/*DS*, biliopancreatic diversion with duodenal switch; *RYGB*, Roux-en-Y gastric bypass; *MAQ*, Moorhead-Ardelt Quality of life Questionnaire. Bold indicates statistical significance

### Biochemical Profile

**Fig. 3:**
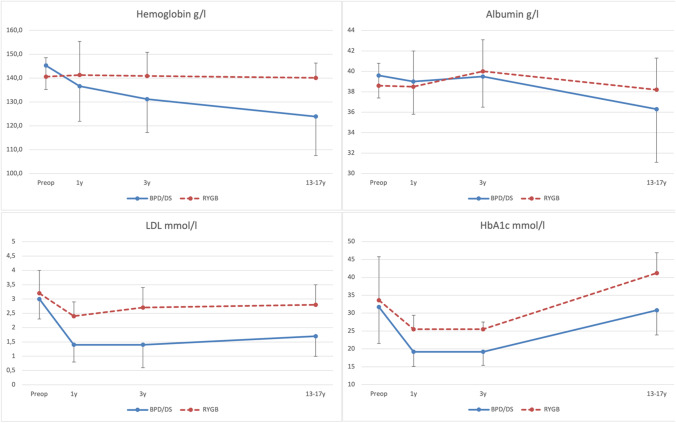
Biochemical profiles. *BPD/DS*, biliopancreatic diversion with duodenal switch; *RYGB*, Roux-en-Y gastric bypass; *Preop*, preoperative; *1y*, 1 year postoperative; *3y*, 3 years postoperative; *13–17y*, 13–17 years postoperative; *LDL*, low density lipoprotein; *HbA1c*, glycated hemoglobin

There was no difference in biochemical profiles at baseline. At 13–17 years postoperatively, the BPD/DS group had lower hemoglobin (122.4 ± 18.8 vs. 140.1 ± 6.2, *p* = 0.002), lower fasting glucose (5.1 ± 0.7 vs. 6.2 ± 0.9, *p* < 0.001), lower HbA1c (30.8 ± 7.1 vs. 41.8 ± 4.9, *p* < 0.001), and lower LDL (1.7 ± 0.7 vs. 2.8 ± 0.7, *p* < 0.001) compared to RYGB. Notably, fasting glucose was higher following RYGB compared to baseline (6.2 ± 0.9 vs. 5.4 ± 0.8, *p* = 0.003). No significant difference in albumin was seen between the two groups (36.3 ± 5.2 vs. 38.2 ± 3.1, *p* = 0.274) (Fig. 3).

### BAROS Score

**Fig. 4:**
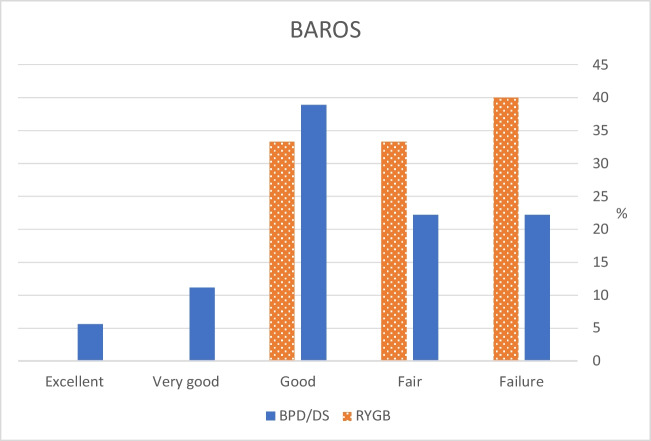
Classification of the overall result based on the total BAROS score. *BAROS*, Bariatric Analysis and Reporting Outcome System; *BPD/DS*, biliopancreatic diversion with duodenal switch; *RYGB*, Roux-en-Y gastric bypass

A superior BAROS score was seen in the BPD/DS group (2.98 vs. 1.34, *p* = 0.047), also with the complication sub-score excluded (3.55 vs. 1.80, *p* = 0.035) (Table 3 and Fig. 4).

## Discussion

This is the first randomized study with follow-up data beyond 10 years comparing RYGB and BPD/DS in patients with super obesity. We could demonstrate that BPD/DS resulted in a sustained superior weight loss, better metabolic control and a superior BAROS score, however, at the cost of a higher rate of nutritional and surgical adverse events.

Superior short-term weight loss in BPD/DS vs. RYGB has been demonstrated in other randomized and non-randomized studies [10, 11, 18, 19, 24]. A slight weight regain is common after all types of bariatric surgery but the fact that half (53.3%) of our RYGB-patients still have a BMI above 40, i.e., still making them candidates for bariatric surgery, is challenging. This problem has been identified by others [25, 26] and is of great concern as a BMI above 40 reduces life expectancy by 8–10 years [27]. Both procedures have well-documented effects on comorbidities. In the present study, with similar rates of diabetes preoperatively, no relapses were found in BPD/DS, while half of the diabetes-free RYGB-patients suffered relapse of their diabetes. Although this finding did not reach statistical significance in this study, superior effect on diabetes has previously been demonstrated by Marinari et al. (100% remission rate) [28] and by Süsstrunk et al. (92.8% complete or partial remission rate) [29], while our former meta-analysis based on 16 studies with single-center comparisons (874 DS and 1149 RYGB) failed to do so [18, 24]. In accordance with other randomized and non-randomized studies, no difference could be seen regarding resolution of hypertension and hyperlipidemia [10, 11, 18, 19, 24].

BPD/DS patients suffered from more adverse effects with an average of 2.7 issues per patient compared to 0.9 with RYGB. Some of the complications were considered severe with one patient requiring reversal surgery due to resistant hypoalbuminemia, two patients requiring emergency surgery due to small bowel perforation and one patient requiring emergency surgery due to perforated diverticulitis. The exact etiology to the bowel perforations is hard to establish, but in one of the small bowel perforations, malnutrition appeared to be a contributing factor. A higher number of consequent surgeries was seen for BPD/DS (0.9 vs. 0.3 surgeries per patient) with cholecystectomy being the most common but failed to reach statistical significance. The increased number of cholecystectomies is presumably due to a combination of higher weight loss and reduced bile re-absorption in BPD/DS. Performing a routine simultaneous cholecystectomy during BPD/DS has been subject to debate, some surgeons have performed it based on surgical judgment while others find it unnecessary [30] as the risk of biliary events is low [31]. It is however notable that 6 out of 18 (33%) BPD/DS-patients later underwent cholecystectomy. The somewhat high incidence of surgically addressed incisional hernias is related to the open approach might not translate well to current practice since 2017 with laparoscopic approach being the method of choice. The higher incidence of complications and subsequent surgeries after BPD/DS have been confirmed in other studies [10, 11, 18, 24]. However, even though the complication rate was higher in BPD/DS, the overall mortality was similar between the groups. Gastrointestinal symptoms were similar between groups except for reflux, which was more common in the BPD/DS group. The increased reflux rate is strongly associated to the gastric sleeve component [32]. Although not reaching statistical significance, we could note that dumping was numerically more common after RYGB, while diarrhea and foul-smelling flatus was seen after BPD/DS, all in accordance with previous studies [18, 19]. We believe that it is important to discuss these differences during preoperative counseling.

Regarding quality of life, the result was similar between the groups even though the BPD/DS group suffered from more adverse effects and gastrointestinal symptoms. We, however, report rather low quality of life scores compared to other studies [33, 34]. Population characteristics such as cultural factors, expectations as well as the long follow-up time, resulting in gradual weight regain might explain these findings.

As expected, the BPD/DS group had superior glucose hemostasis as seen in other studies [10, 18, 19, 24, 35] and increased risk for anemia (12 vs. 4 patients), with a mean hemoglobin reduction of 23 g/l from baseline to the long-term follow up. Although one BPD/DS-patient was reversed due to resistant hypoalbuminemia, no significant difference in albumin was seen between the two groups, both having mean values within the normal range. Hypoalbuminemia and other metabolic deficits, especially in fat-soluble vitamins, are otherwise common after BPD/DS [18, 36]. We have occasionally resorted to use intramuscular cholecalciferol injections in BPD/DS-patients with treatment resistant hypovitaminosis D [37]. Single-Anastomosis Duodenal Switch (SADI-S) had been recently proposed as an alternative to BPD/DS with some authors claiming similar or slightly lower weight loss but also lower morbidity [38–40]. However, more randomized studies are needed to fully elicit the differences.

BAROS is a well-established tool [20], however, seldom used in long-term studies. In the present study, the total BAROS score was still rated “good” or better in 56% of BPD/DS patients compared to only 31% of RYGB patients. The difference is mainly due to the superior weight loss sub-score in BPD/DS. The present average BAROS score (BPD/DS: 2.98 and RYGB: 1.34) is however lower than in other studies, especially those with shorter follow-up. As mentioned previously, we demonstrated a score of 4.7 vs. 4.0 at 4 years postoperatively in a larger cohort of patients having identical care [11], while Patel et al. reported a BAROS score of 3.3 for laparoscopic RYGB at 10 years [34]. At the same time point, Askari et al. demonstrated that 53.2% of LRYGB patients had a “good” or better result [33]. Thus, the BAROS scores seem to decrease with time and some of the differences compared to our study may be attributed to inclusion of patients with less severe obesity, cultural factors affecting quality of life scorings, and observer bias when scrutinizing complications, and differing methodology.

The multiple benefits of BPD/DS outlined above must be weighed against the increased risk of complications and the need for a rigorous follow-up regimen. Some complications are severe and further consume a significant amount of health-care resources aside for patient morbidity. However, relapse of comorbidities and weight gain are as well very problematic as it creates health-related problems further on in life, reducing life expectancy [27]. A two-step approach with an initial sleeve gastrectomy and, in selected cases, addition of the duodenal switch has been proposed by some authors to reduce problems in patients with potential inferior follow up [18, 41]. A second-stage duodenal switch might also be valuable in patients with insufficient weight loss after sleeve gastrectomy, despite having good adherence to the follow-up regimen [41]. Nevertheless, the present long-term results have encouraged us to continue using BPD/DS in our clinical practice.

### Strengths and Limitations

This is the first randomized study comparing RYGB and BPD/DS with follow-up data extending beyond 10 years. Despite the rather small number of patients, we could verify the expected benefits of the two procedures and establish the long-term superiority of BPD/DS concerning BAROS score, weight loss, glucose homeostasis, and HbA1c, at the cost of more complications. However, the small number of patients could make the study under-powered in finding statistical significance for some trends that were noted. Premature closure of inclusion to the study was due to the increasing numbers of patients declining randomization in favor of BPD/DS. Among other potential limitations, the remission of comorbid diseases is somewhat crude as it is based on the use of pharmacological therapy and not exact diagnostic criteria for each disease. We did unfortunately not have access to vitamin and mineral status, nor the cause of death for some of the deceased patients. Weight loss was self-reported which might underestimate the weight at follow-up. However, in a study of 179 overweight bariatric surgery candidates, the BMI misestimation was only negative 0.59% BMI and did not show statistical significance [42].

Moreover, the use of open surgery, standard at our center for patients with super obesity at the time of randomization (2004–2007) explains the rather large number of incisional hernias, while laparoscopic approach, with its well established advantages [43], is standard today since 2016. Furthermore, BAROS scoring can be problematic as the subtraction of points for complications is very observer dependent, and the fact that early postoperative complications lose their significance as time passes. Indeed, several authors have raised critique regarding this instrument [44]. Considering that the operated population ages with time, and in turns develops comorbidities as the general populations does, the comorbidity scores will unenviably migrate toward failure. Some trends could be noted regarding the comorbidities, especially diabetes mellitus, but unfortunately, this study lacked power to show statistical significance.

## Conclusion

In conclusion, we could verify that BPD/DS results in superior weight loss and metabolic control, at the cost of more complications, when compared to RYGB in patients with super obesity at 13–17 years after surgery. In addition, the global assessment tool BAROS demonstrated superior scores in the BPD/DS group.

## Data Availability

Aggregated data supporting this study are available upon reasonable request. Please contact filip.moller@surgsci.uu.se.
